# Oxygen conduction mechanism in Ca_3_Fe_2_Ge_3_O_12_ garnet-type oxide

**DOI:** 10.1038/s41598-019-39288-x

**Published:** 2019-02-22

**Authors:** Joohwi Lee, Nobuko Ohba, Ryoji Asahi

**Affiliations:** Toyota Central R&D Laboratories, Inc., Nagakute, Aichi 480-1192 Japan

## Abstract

We investigate the oxygen conduction mechanism in a garnet-type oxide, Ca_3_Fe_2_Ge_3_O_12_, for the first time in detail by first-principle calculations. The nudged elastic band results confirm that this oxide has a lower migration barrier energy (0.45 eV) for an oxygen interstitial (O_*i*_) with the kick-out mechanism than that (0.76 eV) for an oxygen vacancy. The migration paths for O_*i*_ are delocalized and connected to the neighboring cells in three-dimensional space. This oxide does not have a very low formation energy of O_*i*_ when the Fermi level is near the lowest unoccupied molecular orbital at a high temperature, which implies the possibility of electron doping by high-valence cations. These theoretical results suggest that the doping of Ca_3_Fe_2_Ge_3_O_12_ for generation of excess O_*i*_ provides a good oxygen-ion conductivity, along with the electronic conductivity.

## Introduction

Oxygen-ion conductors with high oxygen-ion conductivities (*σ*_O_) have been developed owing to their desirable characteristics for applications as electrolytes of solid oxide fuel cells (SOFCs), oxygen separation membranes, and gas sensors^1,2^. Nowadays, Y-doped (-stabilized) ZrO_2_ (YSZ) is mostly used owing to its advantages such as abundance, chemical stability, nontoxicity, and low cost. It exhibits *σ*_O_ of ~10^−2^ S/cm at a high temperature (*T*) of ~1000 K (ref.^3^). In order to improve the industrial applicability, it is necessary to decrease its *T* while maintaining *σ*_O_. Some oxides such as Gd-doped CeO_2_ (GDC)^4^, pure or Er-doped *δ*-phase of Bi_2_O_3_^5,6^, and doped LaGaO_3_^7–11^, have higher *σ*_O_ values at the same *T* than that of YSZ. However, a new type of oxygen-ion conductor with sufficient merits that can substitute YSZ is required.

Garnet-type oxides such as Li_7_La_3_Zr_2_O_12_ (LLZO) have been reported as promising lithium-ion conductors^12–16^. This oxide has a high Li-ion conductivity when the cubic crystal structure is achieved in high temperature or stabilized by extrinsic dopants such as Nb and Ta. Partial occupancies of Li in unstable sites and formation of threedimensional channels are ascribed to high Li-ion conductivity^14,15^. To the best of our knowledge, oxygen-ion conductions for the garnet-type oxides have not been widely investigated. Kubicek *et al*.^16^, recently just reported that the oxygen-ion diffusivity in Ta-doped LLZO measured by isotope exchange experiment is comparable to the value in YSZ at 350 °C. Therefore, it is worth investigating an applicability of garnet-type oxides as oxygen-ion conductors.

It is necessary to know the properties relevant to *σ*_O_ of a fundamental crystal structure, namely, normal garnet crystal structure with the composition of *A*_3_(^2+^)*B*_2_(^3+^)*C*_3_(^4+^)O_12_. We choose one of the stable garnet-type oxides, Ca_3_Fe_2_Ge_3_O_12_. This oxide could be grown as a single crystal by the flux method^17^. First-principle databases such as Materials Project Database (MPD)^18^ suggest that this oxide forms the convex-hull state without separation into the mixture of other oxides. We investigated two important properties related to *σ*_O_, namely, formation energy (*E*_*form*_) and migration barrier energy (*E*_*mig*_) for both oxygen vacancy (*V*_O_) and oxygen interstitial (O_*i*_), in the Ca_3_Fe_2_Ge_3_O_12_ garnet-type oxide by first-principles calculations to clarify its oxygen-conduction mechanism.

## Results and Discussion

### Garnet crystal structure of Ca_3_Fe_2_Ge_3_O_12_

**Figure 1:**
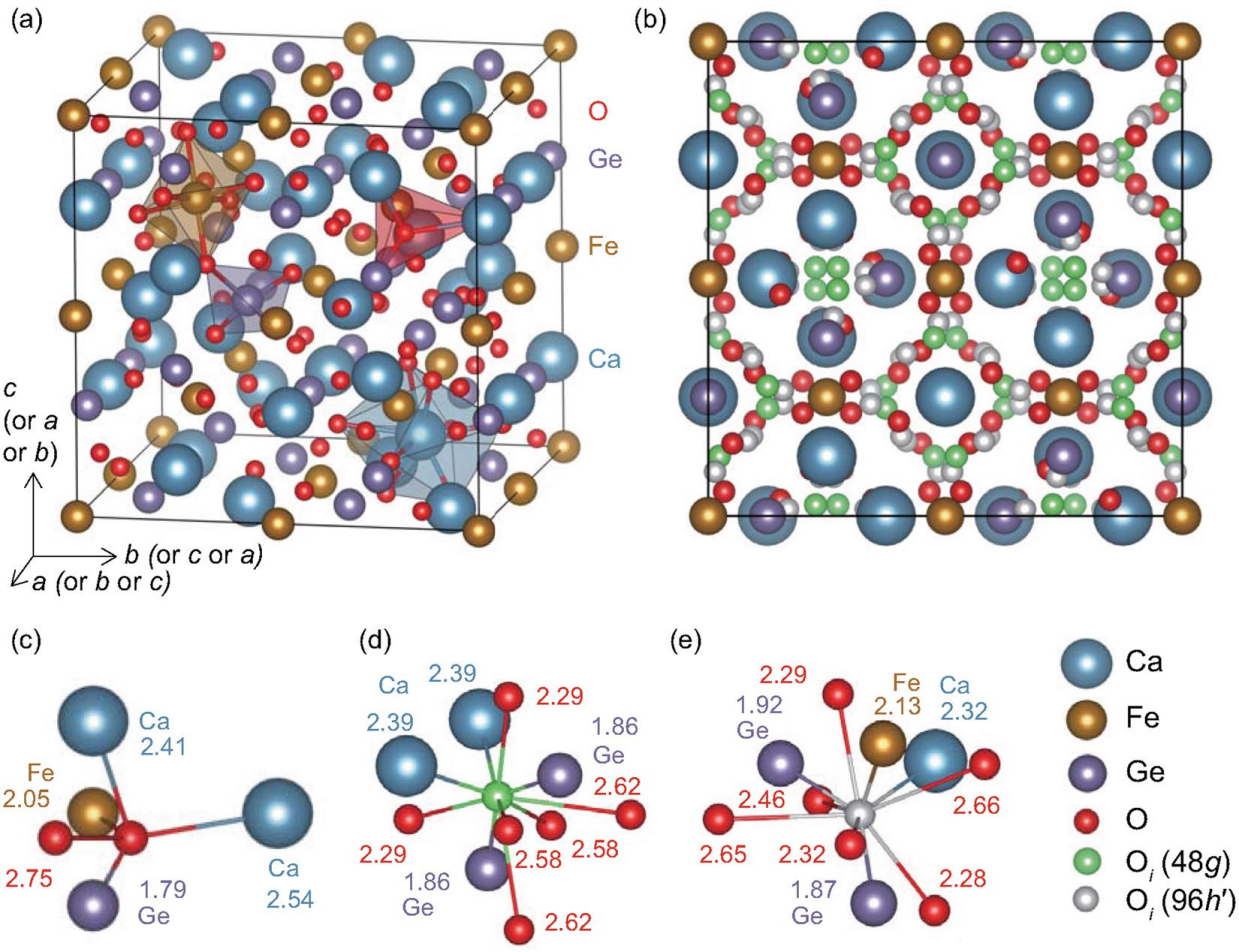
(**a**) Garnet crystal structure (space group: *Ia*-3*d*) of Ca_3_Fe_2_Ge_3_O_12_; the conventional cell with the cubic structure (Ca_24_Fe_16_Ge_24_O_96_, 160 atoms) is shown. (**b**) Distribution of equivalent 48*g* and 96*h*′ sites after the optimization of the internal coordinates with one doubly charged O_*i*_. The internal coordinates of the cations and O are equal to those of the perfect crystal; only the changes in the internal coordinates of O_*i*_ are shown in the optimized cells. In the calculations, one O_*i*_ is incorporated in the computational cell. Nearest-neighboring atoms of (**c**) O in the perfect crystal, (**d**) O_*i*_ in the 48*g* site, and (**e**) O_*i*_ in the 96*h*′ site in the optimized structure including one O_*i*_. The numerical values are the distances (bond lengths), expressed in Å. The cutoff radius for the nearest-neighboring atoms in the figure is 2.8 Å, which is ~10% longer than the longest Ca–O bond.

Figure 1 shows the garnet crystal structure of Ca_3_Fe_2_Ge_3_O_12_ belonging to the cubic crystal system with a space group of *Ia*-3*d*. Table 1 summarizes the internal coordinates, multiplicities, and Wyckoff positions of sites occupied by constituent chemical elements. The effective coordination numbers of Fe and Ge are natural numbers, 6 (in an octahedral site) and 4 (in a tetrahedral site), respectively, whereas that of Ca is ~7.8 [in a dodecahedral site (bisdisphenoid)]. The bond lengths of Fe–O and Ge–O significantly differ (2.05 and 1.79 Å, respectively). The Ca–O bond lengths, which are significantly longer than the above two lengths, slightly deviate; half of them are 2.41 Å, while the other half are 2.54 Å. All of the O atoms occupy the same sites, 96*h*, forming two, one, and one chemical bonds (in the tetrahedral site) with Ca, Fe, and Ge, respectively. Therefore, the *V*_O_ sites are identical to each other.

**Table 1:** Internal coordinates of the constituent elements in the perfect crystal and initial internal coordinates of the O_*i*_ sites of the garnet-type Ca_3_Fe_2_Ge_3_O_12_ with the cubic crystal structure (160 atoms) before the optimization of the internal coordinates.

Chemical element	Type	Site multiplicity	Wyckoff letter	*x*	*y*	*z*
Ca (*A*^2+^ cation)	Perfect crystal	24	*c*	1/4	1/8	0
Fe (*B*^3+^ cation)	Perfect crystal	16	*a*	0	0	0
Ge (*C*^4+^ cation)	Perfect crystal	24	*d*	1/2	1/4	1/8
O	Perfect crystal	96	*h*	0.2834	0.0979	0.1989
O_*i*_ (16*b*)	Additional O_*i*_	16	*b*	1/8	1/8	1/8
O_*i*_ (32*e*)	Additional O_*i*_	32	*e*	0.1705	0.3295	0.6705
O_*i*_ (48*g*)	Additional O_*i*_	48	*g*	0.8750 (0.8750)^b^	0.2704 (0.2734)^b^	0.4796 (0.4766)^b^
O_*i*_ (96*h*′)^a^	Additional O_*i*_	96	*h*	0.9167 (0.9074)^b^	0.3618 (0.3319)^b^	0.5368 (0.4856)^b^

The theoretical lattice parameter for this cubic crystal structure is 12.48 Å.

^a^In order to distinguish the Wyckoff letter of this O_*i*_ site from that of the O site in the perfect crystal, we denote this O_*i*_ site as 96*h*′.

^b^The internal coordinates of the O_*i*_ site with one O_*i*_^2−^ after the relaxations are shown in the parentheses.

We consider several types of O_*i*_ sites. The initial positions of the O_*i*_ sites in the computations are summarized in Table 1, which were considered by an empty-space-finder module implemented in the MedeA program^19^. Among the four types of O_*i*_ sites, we focus on two sites, 48*g* and 96*h*′, which are energetically stable and lead to satisfactory computational convergences. These O_*i*_ sites are shown in Figs 1 and Supplementary S1. The other two sites, 16*b* and 32*e*, are not analyzed in detail in this study, as we considered that they are not energetically preferred. When a neutral (O_*i*_^0^) and doubly charged oxygen interstitials (O_*i*_^2−^) occupy the 16*b* sites, the corresponding *E*_*form*_ values are 0.66 and 1.04 eV higher than those of the case where O_*i*_ occupy the most stable sites, respectively. The calculations with O_*i*_ in the 32*e* sites were not well converged, leading to a significantly higher energy and inability to satisfy the optimization condition.

O_*i*_ in the 48*g* site participates in chemical bonds with two Ca and two Ge, whereas O_*i*_ in the 96*h*′ site participates in chemical bonds with one Ca, one Fe, and two Ge. The bond lengths are optimized to be closer to their optimal values in the perfect crystal structure after relaxation of their internal coordinates. The Ca–O_*i*_ bond length is slightly shorter than the Ca–O bond length in the perfect crystal, whereas the Ge–O_*i*_ and Fe–O_*i*_ bond lengths are slightly longer than the Ge–O and Fe–O bond lengths in the perfect crystal, respectively. In addition, O_*i*_ in the 48*g* or 96*h*′ sites participate in several bonds with O. The O_*i*_–O bond lengths are generally longer than the cation–O bond lengths and shorter than the O–O distance in the perfect crystal structure, but some of them are similar to the Ca–O bond length.

### *E*_*mig*_ of an oxygen defect in Ca_3_Fe_2_Ge_3_O_12_

Regarding the oxygen-ion conduction, to the best of our knowledge, it is still not clear whether *V*_O_ or O_*i*_ is the dominant oxygen defect in the garnet-type oxides. In order to compare the migration barrier energies of *V*_O_ and O_*i*_, we employ the climbing image nudged elastic band (CI-NEB) method^20,21^ for several candidates of migration paths. As the migrating oxygen defects, a doubly charged oxygen vacancy (*V*_O_^2+^) and O_*i*_^2−^ are employed.

**Figure 2:**
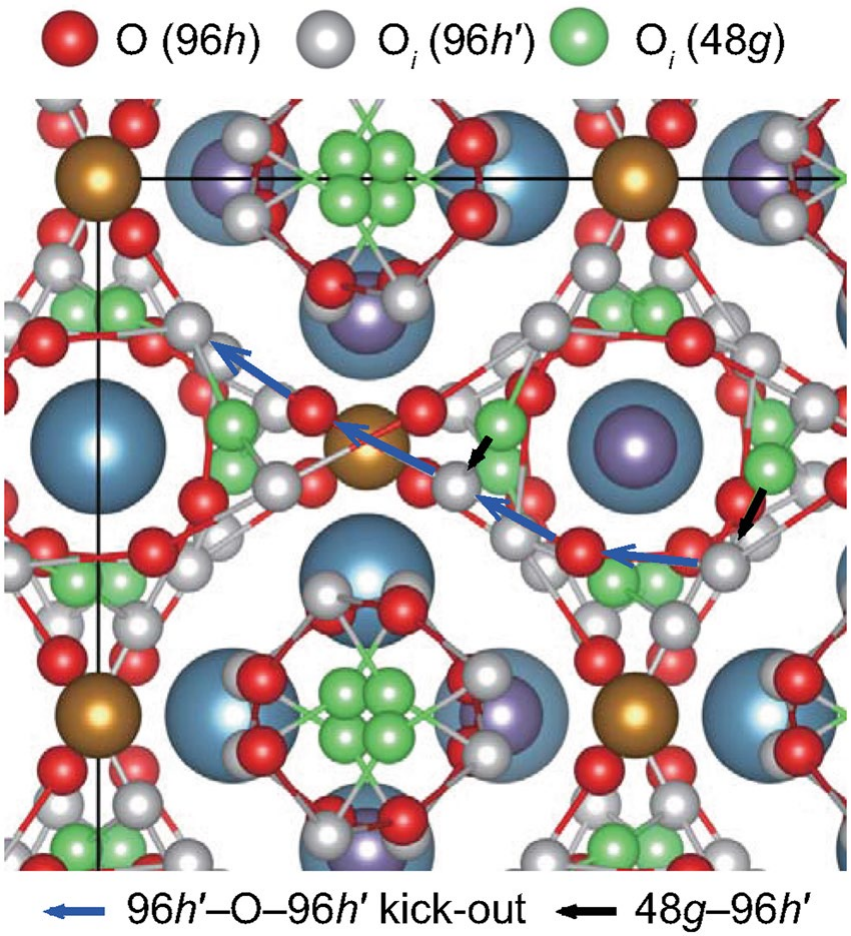
Two main migration paths of O_*i*_ and two types of equivalent O_*i*_ (48*g* and 96*h*′) sites in the perfect crystal of the garnet-type Ca_3_Fe_2_Ge_3_O_12_. A part of the conventional cell of Fig. 1 is presented, showing all of the equivalent O_*i*_ sites. The colors representing the cations are the same as those in Fig. 1. The 96*h*′–O–96*h*′ migration path with the kick-out mechanism is delocalized and connected to the neighboring cells in three-dimensional space.

**Figure 3:**
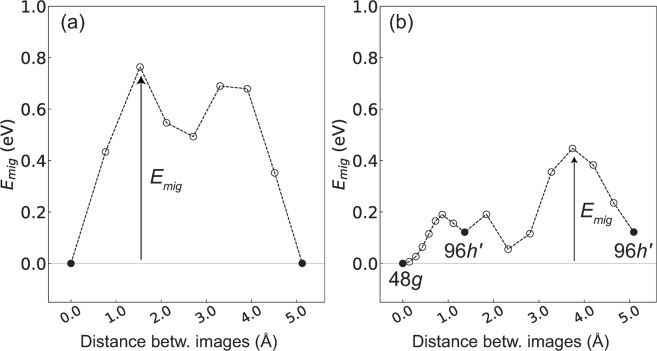
*E*_*mig*_ for (**a**) *V*_O_ and (**b**) O_*i*_ in the garnet-type Ca_3_Fe_2_Ge_3_O_12_. The oxygen defects are doubly charged. The closed circles denote the initial or final states, fixed in the CI-NEB method. Two migration paths for O_*i*_, namely, the 48*g*–96*h*′ direct migration and 96*h*′–O–96*h*′ with the kick-out mechanism, are shown in Fig. 2.

We show the investigated migration paths with *E*_*mig*_ among various migration paths in Supplementary Figs. S2 (for *V*_O_^2+^) and S3 (for O_*i*_^2−^). Two migration paths for O_*i*_ with low *E*_*mig*_ values among the various migration paths are considered in Fig. 2. One of them is the migration path between the 48*g* and 96*h*′ sites with direct migration of O_*i*_ (48*g*–96*h*′), while the other is the migration path between two 96*h*′ sites with the kick-out mechanism (96*h*′–O–96*h*′). Between the 48*g* and 96*h*′ sites, the 48*g* site is more favorable to be occupied by O_*i*_^2−^. However, the distance between two 48*g* sites is as far as 3.16 Å, while the distance between 48*g* and 96*h*′ sites is only 1.44 Å. This implies that direct migration of O_*i*_ can occur from the 48*g* site to the 96*h*′ site with a low *E*_*mig*_. Compared with the distance between the two 48*g* sites, the shortest distance between two 96*h*′ sites is only 2.26 Å. In addition, another type of migration path based on the kick-out mechanism can exist. One O_*i*_ in the 96*h*′ site moves to the O site; the kicked-out O atom moves to another O_*i*_ in the 96*h*′ site. In this case, the distance between the 96*h*′ site and O in the 96*h* site is only 1.9 Å. Combining the two migration paths, we suggest a long migration path between two 48*g* sites passing through the 96*h*′ sites, as shown in Fig. 3(b). The 96*h*′–O–96*h*′ migration path is delocalized and connected in the whole cell. If the 96*h*′ site is more stable than the 48*g* site for O_*i*_, the 48*g*–96*h*′ migration path, which is localized, may not be needed to be considered for the lowest *E*_*mig*_.

There is another migration path with a low *E*_*mig*_ (only 0.19 eV, 96*h*′–48*g*–96*h*′ migration path) between two 96*h*′ sites through the 48*g* site, as shown in Supplementary Fig. S3(c). However, as shown in Supplementary Fig. S4, this migration path is the same as the 48*g*–96*h*′ migration path and is localized, so that the migration of O_*i*_ through the whole cell cannot occur only through this migration path. The 96*h*′–O–96*h*′ migration path is additionally needed to connect through the whole cell, as mentioned above.

Figure 3 shows the lowest *E*_*mig*_ obtained by the CI-NEB method among those of the considered migration paths for *V*_O_ and O_*i*_. The intermediate procedures of movement of O_*i*_ along the 48*g*–96*h*′ and 96*h*′–O–96*h*′ migration paths are shown in Supplementary Figs S5 and S6. *E*_*mig*_ for O_*i*_ is significantly lower (0.45 eV) than that for *V*_O_ (0.76 eV). In addition, for the migration of *V*_O_ through the whole cell, *E*_*mig*_ higher than 0.76 eV with several combinations of migration paths is necessary because the migration path with *E*_*mig*_ of 0.76 eV is localized and not connected through the whole cell. This result suggests that the oxygen-ion conduction based on O_*i*_ with a significantly lower *E*_*mig*_ of 0.45 eV may be dominant. Therefore, the extrinsic doping with aliovalent cations with higher valences to make O_*i*_ rather than *V*_O_ can be useful to increase *σ*_O_ of Ca_3_Fe_2_Ge_3_O_12_.

### *E*_*form*_ of an oxygen defect in Ca_3_Fe_2_Ge_3_O_12_

**Figure 4:**
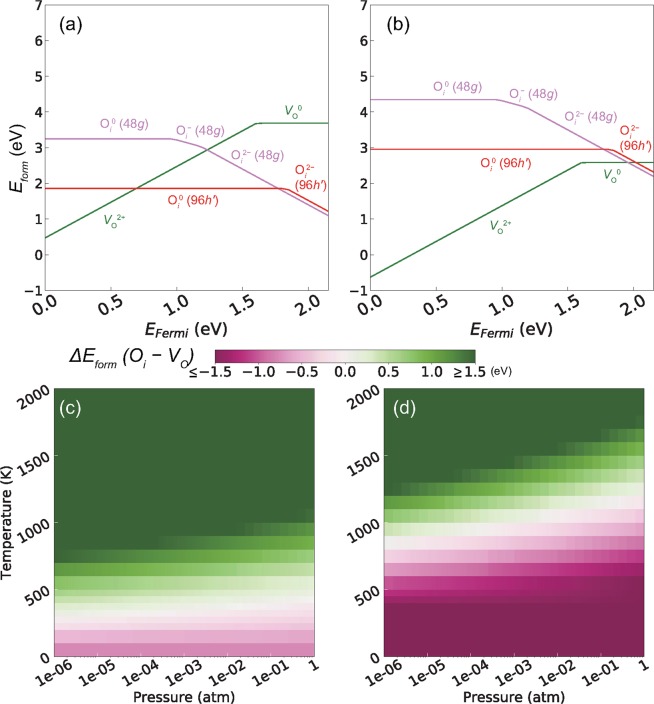
*E*_*form*_ of *V*_O_ and O_*i*_ as a function of *E*_*Fermi*_ at the (**a**) O-rich condition and (**b**) *p* of 1 atm and *T* of 1000 K for the garnet-type Ca_3_Fe_2_Ge_3_O_12_. Δ*E*_*form*_(O_*i*_–*V*_O_) as a function of *p* and *T* when *E*_*Fermi*_ is (**c**) at the center of the band-gap and (**d**) at the LUMO. A negative Δ*E*_*form*_(O_*i*_–*V*_O_) value implies that O_*i*_ preferentially forms compared to *V*_O_.

It is worth investigating the formation condition of O_*i*_ utilizing its low *E*_*mig*_. It is well known that *E*_*form*_ of an oxygen defect in an oxide compound strongly depends on the variables in equation (1) (see Method section.) such as the charge of the point defect (*q*), Fermi level (*E*_*Fermi*_), and chemical potential of O (*μ*_O_) depending on the pressure (*p*) and *T* (refs^22–25^). Figure 4(a) shows *E*_*form*_ of *V*_O_ and O_*i*_ as a function of *E*_*Fermi*_ at the O-rich condition (*μ*_O_ = half-energy of O_2_). When *E*_*Fermi*_ is near the center of the band-gap [the band-gap of the garnet-type Ca_3_Fe_2_Ge_3_O_12_ obtained by the generalized gradient approximation (GGA) + U method is 2.15 eV], the neutral O_*i*_^0^ is the most stable. When *E*_*Fermi*_ is shifted to a position near the highest occupied molecular orbital (HOMO, or valence band maximum), *V*_O_^2+^ is stabilized (from the HOMO to 0.70 eV over the HOMO). When *E*_*Fermi*_ is shifted to the lowest unoccupied molecular orbital (LUMO, or conduction band minimum), O_*i*_^2−^ is stabilized (from 1.77 eV over the HOMO to the LUMO). This result implies that O_*i*_ can be easily generated at the O-rich condition.

However, more realistic condition at a higher *T* should be considered for oxide synthesis and oxygen-ion conductor applications. Figure 4(b) shows *E*_*form*_ of *V*_O_ and O_*i*_ as a function of *E*_*Fermi*_ at *p* = 1 atm and *T* = 1000 K, which were selected based on the condition for the applications. *μ*_O_ is obtained as the half-energy of O_2_ minus 1.1 eV^23,26^. When *T* increased to 1000 K, *V*_O_^2+^ or *V*_O_^0^ stabilized for most of the *E*_*Fermi*_ values. The *E*_*Fermi*_ range for stable O_*i*_^2−^ at this *T* is only from 0.20 eV below the LUMO to the LUMO.

In order to reveal the thermodynamic stabilities of *V*_O_ and O_*i*_ in terms of *p* and *T*, we plot the difference in *E*_*form*_ between *V*_O_ and O_*i*_ [Δ*E*_*form*_(O_*i*_ − *V*_O_)] at a fixed *E*_*Fermi*_. Figure 4(c) shows Δ*E*_*form*_(O_*i*_ − *V*_O_) when *E*_*Fermi*_ is at the center of the band-gap. When *p* is 1 atm, the *T* range for more stable O_*i*_ than *V*_O_ is below ~450 K. Figure 4(d) shows Δ*E*_*form*_(O_*i*_ − *V*_O_) when *E*_*Fermi*_ is at the LUMO. Compared with the former condition, *T* for stable O_*i*_ increased to ~1100 K. However, it may not be sufficiently high because the usual sintering *T* for the oxides is higher than 1000 K. In addition, regardless of the location of *E*_*Fermi*_, the stabilizing *T* range for O_*i*_ generally decreases with the decrease in *p*. Therefore, in order to increase the O_*i*_ content, a high *p* for the oxygen-gas sources is required.

This result implies that O_*i*_ cannot be easily generated in Ca_3_Fe_2_GeO_12_ despite its high potential for application as an oxygen-ion conductor. Processing at a lower *T* than ~1000 K may be necessary to provide O_*i*_ in Ca_3_Fe_2_GeO_12_ by doping of aliovalent cations with higher valences than those of Ca, Fe, and Ge and thus increase *E*_*Fermi*_ to a level near the LUMO. However, the doped cations can also act as “electron donors”. When positively charged defects such as La_Ca_^+^ are introduced by extrinsic doping, negative charges such as O_*i*_^2−^ (for semiconductors, negatively charged defects are sometimes referred to as “electron killers^27^”) and electrons are necessary to compensate for the charge imbalance. Therefore, a high *E*_*form*_ of O_*i*_^2−^ implies the possibility of electron doping considering the charge neutrality requirement.

In fact, our research group recently confirmed by a prediction model based on the machine learning technique and post-experiment that a La-doped Ca_3_Fe_2_Ge_3_O_12_ (Ca_2.7_La_0.3_Fe_2_Ge_3_O_12+*δ*_) with the garnet crystal structure has a good oxygen-ion conduction^28^. However, its ionic transport number of *σ*_O_ in the total conductivity (~10^−2^ S/cm at 700 °C) was ~10%; therefore, the electronic conductivity was also observed. Considering theoretical results in this study and experimental results of ref.^28^, we expect that both of excess O_*i*_ and electrons can be generated as transport carriers in Ca_3_Fe_2_Ge_3_O_12_ with doping of aliovalent cations with higher valences than those of Ca, Fe, and Ge.

As *E*_*form*_ of O_*i*_^2−^ decreases with a gradient of −2, a lower *E*_*form*_ of O_*i*_^2−^ near the LUMO can be achieved by increasing the band-gap. This suggests that band-gap engineering by mixing the constituent elements with others with the same valences can increase the band-gap, as in previous studies on In_*x*_Ga_1−*x*_N^29^, mixed anion lead halide perovskites^30^, and Ni- and Co-doped ZnO nanoparticles^31^. In the In_*x*_Ga_1−*x*_N case, the LUMO increases as an increase of the Ga content because an energy level of Ga *s* is higher than that of In *s*, resulting in an increase of the band-gap. In the present case, Fe 3*d* forms the bottom of the LUMO, significantly below the unoccupied *s* levels of the other cations, as shown in Supplementary Fig. S7. Therefore, partial substitutions of trivalent cations, which can shift up the location of the LUMO from the Fe 3*d* levels, for Fe may be effective to increase the band-gap.

Finally, we calculated the formation energies of antisites (atomic interchange, a pair of *X*_*Y*_ and *Y*_*X*_), which can be obtained by the differences in energies between supercells with and without antisites. The positions of the antisites were obtained by exchanging nearest-neighboring sites under the stoichiometric condition. The generated antisites were not very stable. For most of the antisites, the energies increased by more than 1 eV, as shown in Supplementary Table 1. When Fe and O were exchanged, the structure became unstable, so that it was optimized to a perfect crystal without antisites. The high formation energies of cation antisites are reasonable because the local environments of the three types of cation sites are different in the garnet crystal structure^32^. The high formation energies of the cation antisites are also in agreement with the experimental result that Ca_3_Fe_2_Ge_3_O_12_ prefers to form the normal garnet crystal structure without inversion (exchange of cation sites)^17^. Moreover, the formation energies of oxygen antisites were larger than 5 eV, which indicates that oxygen cannot be easily incorporated in cation sites.

In summary, we investigated the oxygen-ion conduction mechanism in the Ca_3_Fe_2_Ge_3_O_12_ garnet-type oxide by the first-principles calculations. Ca_3_Fe_2_Ge_3_O_12_ exhibited a low *E*_*mig*_ of O_*i*_ of 0.45 eV with the kick-out mechanism. The migration path with this low *E*_*mig*_ was delocalized and connected to the neighboring cells in three-dimensional space. In addition, this value was lower than that of *V*_O_ of 0.76 eV. However, this oxide had a shallow range of *E*_*Fermi*_ just below the LUMO to form O_*i*_ at a practical *T* of 1000 K. This implies that high concentrations of extrinsic dopants, which may act as electron donors, are needed. Therefore, we expect that processing at a low *T* and band-gap engineering to achieve a larger band-gap and thus generate stable O_*i*_ are needed to achieve and improve *σ*_O_ of this oxide.

The findings in this study provide valuable insights, which can inspire further investigations on the garnet-type oxygen-ion conductors. The proposed computational strategy can be employed for analyses of other combinations with this crystal structure.

## Method

All of the first-principles calculations were performed using the projector augmented wave (PAW)^33,34^ method implemented in the Vienna *Ab-initio* Simulation Package (VASP)^35,36^. We used the GGA exchange–correlation functional parameterized in the Perdew–Burke–Ernzerhof (PBE) form^37^ along with the on-site Coulomb interaction^38^ with an effective *U*−*J* of 4.3 eV (GGA + U) for the *d*-orbitals of Fe. This was implemented because the band-gap obtained by the GGA method was only 0.1 eV. Owing to the lack of references for Ca_3_Fe_2_Ge_3_O_12_, the value of *U*−*J* was selected using previous theoretical calculations for *α*-Fe_2_O_3_^24,39^, which has the same valence of Fe^3+^. Mosey *et al*.^39^ proposed a reasonable *U*−*J* value, which converged well with the increase in the computational unit of *α*-Fe_2_O_3_, and confirmed that GGA+U with *U*−*J* of 4.3 eV was in good agreement with the lattice parameter, volume, bulk modulus, and band-gap obtained in experiments. We confirmed that the lattice parameter (12.48 Å) of Ca_3_Fe_2_Ge_3_O_12_ obtained by GGA+U is in good agreement with the corresponding value (12.32 Å) obtained in the experiment^17^.

The structural relaxations of the primitive cells (80 atoms) with the associated changes in lattice constants and atomic coordinates were performed until the interatomic force on each atom was reduced below 0.005 eV/Å. The cutoff energy was set to 500 eV. The Brillouin zone was sampled using Γ-centered 4 × 4 × 4 meshes. Brillouin zone integrations were performed using Gaussian smearing with a smearing width of 0.1 eV. Electron spin polarizations were turned on.

We also computed *E*_*form*_ and *E*_*mig*_ as their low values are favorable for a higher *σ*_O_^40,41^. The *E*_*form*_ values were computed using the conventional cubic cell (160 atoms with a lattice constant larger than 10 Å)^22^:

1 $${E}_{form}^{q}=E({{\rm{Ca}}}_{3}{{\rm{Fe}}}_{2}{{\rm{Ge}}}_{3}{{\rm{O}}}_{12}:{D}^{q})-E({{\rm{Ca}}}_{3}{{\rm{Fe}}}_{2}{{\rm{Ge}}}_{3}{{\rm{O}}}_{12})-{n}_{D}{\mu }_{O}+q({E}_{{\rm{HOMO}}}+{E}_{Fermi})+{\rm{\Delta }}{E}_{{\rm{LZ}}},$$

where *E*(Ca_3_Fe_2_Ge_3_O_12_:*D*^*q*^) is the energy of a supercell including a point defect (*V*_O_ or O_*i*_), *E*(Ca_3_Fe_2_Ge_3_O_12_) is the energy of a supercell of the perfect Ca_3_Fe_2_Ge_3_O_12_, *n*_*D*_ is 1 (or −1), which corresponds to the interstitial defect (or vacancy), *μ*_O_ is the chemical potential of an added (or removed) O, *q* is the charge of the point defect, *E*_HOMO_ is the eigenvalue of the HOMO formed mainly by O 2*p*, *E*_*Fermi*_ is the Fermi level as a variable, and Δ*E*_LZ_ is the correction term proposed by Lany and Zunger^42,43^ to compensate the image charge interactions between supercells for the charged point defect. The calculations using the supercell including a defect were performed until the interatomic forces on each atom were reduced below 0.02 eV/Å at a fixed lattice constant. Only the Γ-point was used for the ***k***-space sampling. The computed energy differences between the primitive and conventional cells were below ~1 meV/atom. The transformations between the primitive and conventional cells were performed by SPGLIB^44^ implemented in PHONOPY^45,46^. The dielectric constant (13.0 for Ca_3_Fe_2_Ge_3_O_12_) for the correction term of the image charge interaction^42,43^ was computed using the primitive cell based on the density functional perturbation theory^47^. The supercells with point defects were created by removing or adding twice more electrons compared to the number of removed or added oxygen atoms to compensate the background charges. For the neutral O_*i*_, the nonmagnetic state (compensated spin polarization) was used as its energy was significantly lower (more than 1 eV) than that of the ferromagnetic state (magnetic moment of 2).

The *E*_*mig*_ values were calculated using the CI-NEB method^20,21^ with three intermediate images. When the convergence of the computation was not satisfactory or when more detailed migration paths should be investigated, seven intermediate images were employed. The CI-NEB calculations were performed until the forces decreased below 0.03 eV/Å with a spring constant of 5 eV/Å^2^ between the images. We employed a doubly charged oxygen vacancy (*V*_O_^2+^) and oxygen interstitial (O_*i*_^2−^) for *E*_*mig*_ assuming that these charged point defects were formed by extrinsic doping with aliovalent cations. Visualizations of the crystal structures and confirmation of the effective coordination numbers of the cations were performed using the VESTA program^48^.

## Supplementary information

Supplement

## References

[1] Knauth P, Tuller HL (2002). Solid‐state ionics: roots, status, and future prospects. J. Am. Ceram. Soc..

[2] Skinner SJ, Kilner JA (2003). Oxygen ion conductors. Mater. Today.

[3] Gellings, P. J. & Bouwmeester, H. Handbook of solid state electrochemistry (CRC press, 1997).

[4] Mogensen M, Sammes NM, Tompsett GA (2000). Physical, chemical and electrochemical properties of pure and doped ceria. Solid State Ion..

[5] Sammes N, Tompsett G, Näfe H, Aldinger F (1999). Bismuth based oxide electrolytes—structure and ionic conductivity. J. Eur. Ceram. Soc..

[6] Shitara K (2017). First-Principles selection of solute elements for Er-Stabilized Bi_2_O_3_ oxide-ion conductor with improved long-term stability at moderate temperatures. Chem. Mater..

[7] Ishihara T, Matsuda H, Takita Y (1994). Doped LaGaO_3_ perovskite type oxide as a new oxide ionic conductor. J. Am. Chem. Soc..

[8] Drennan J (1997). Characterisation, conductivity and mechanical properties of the oxygen-ion conductor La_0.9_Sr_0.1_Ga_0.8_Mg_0.2_O_3-x_. J. Mater. Chem..

[9] Huang K, Goodenough JB (2000). A solid oxide fuel cell based on Sr- and Mg-doped LaGaO_3_ electrolyte: the role of a rare-earth oxide buffer. J. Alloys Compd..

[10] Ishihara T, Shibayama T, Honda M, Nishiguchi H, Takita Y (2000). Intermediate temperature solid oxide fuel cells using LaGaO_3_ electrolyte II. Improvement of oxide ion conductivity and power density by doping Fe for Ga site of LaGaO_3_. J. Electrochem. Soc..

[11] Gao Z, Miller EC, Barnett SA (2014). A high power density intermediate‐temperature solid oxide fuel cell with thin (La_0.9_Sr_0.1_)_0.98_(Ga_0.8_Mg_0.2_)O_3‐δ_ electrolyte and nano‐scale anode. Adv. Func. Mater..

[12] Kokal I, Somer M, Notten P, Hintzen H (2011). Sol–gel synthesis and lithium ion conductivity of Li_7_La_3_Zr_2_O_12_ with garnet-related type structure. Solid State Ion..

[13] Meier K, Laino T, Curioni A (2014). Solid-state electrolytes: revealing the mechanisms of Li-ion conduction in tetragonal and cubic LLZO by first-principles calculations. J. Phys. Chem. C.

[14] Jalem R (2013). Concerted migration mechanism in the Li ion dynamics of garnet-type Li_7_La_3_Zr_2_O_12_. Chem. Mater..

[15] Miwa K, Asahi R (2018). Molecular dynamics simulations with machine learning potential for Nb-doped lithium garnet-type oxide Li7−xLa3(Zr2−xNbx)O12. Phys. Rev. Mater..

[16] Kubicek M (2017). Oxygen vacancies in fast lithium-ion conducting garnets. Chem. Mater..

[17] Lévy D, Barbier J (1999). Normal and inverse garnets: Ca_3_Fe_2_Ge_3_O_12_, Ca_3_Y_2_Ge_3_O_12_ and Mg_3_Y_2_Ge_3_O_12_. Acta Crystallogr. C.

[18] Jain A (2013). Commentary: The Materials Project: A materials genome approach to accelerating materials innovation. Appl. Phys. Lett. Mater..

[19] Materials Design, Inc. MedeA. https://www.materialsdesign.com/medea (2018).

[20] Henkelman G, Uberuaga BP, Jónsson H (2000). A climbing image nudged elastic band method for finding saddle points and minimum energy paths. J. Chem. Phys..

[21] Henkelman G, Jónsson H (2000). Improved tangent estimate in the nudged elastic band method for finding minimum energy paths and saddle points. J. Chem. Phys..

[22] Van de Walle CG, Neugebauer J (2004). First-principles calculations for defects and impurities: Applications to III-nitrides. J. Appl. Phys..

[23] Reuter K, Scheffler M (2001). Composition, structure, and stability of RuO_2_ (110) as a function of oxygen pressure. Phys. Rev. B.

[24] Lee J, Han S (2013). Thermodynamics of native point defects in α-Fe_2_O_3_: an *ab initio* study. Phys. Chem. Chem. Phys..

[25] Oba F, Togo A, Tanaka I, Paier J, Kresse G (2008). Defect energetics in ZnO: A hybrid Hartree-Fock density functional study. Phys. Rev. B.

[26] Stull, D. R. & Prophet, H. JANAF thermochemical tables (1971).

[27] Zunger A (2003). Practical doping principles. Appl. Phys. Lett..

[28] Kajita, S., Ohba, N., Suzumura, A., Tajima, S. & Asahi, R. Discovery of superionic conductors by ensemble-scope descriptor. Submitted. (2018).

[29] Davydov VY (2002). Band gap of InN and In-rich In_x_Ga_1—x_N alloys (0.36 < x < 1). Phys. Status Solidi B.

[30] Kulkarni SA (2014). Band-gap tuning of lead halide perovskites using a sequential deposition process. J. Mater. Chem. A.

[31] Ali RN (2018). Band gap engineering of transition metal (Ni/Co) codoped in zinc oxide (ZnO) nanoparticles. J. Alloys Compd..

[32] Ye W, Chen C, Wang Z, Chu I-H, Ong SP (2018). Deep neural networks for accurate predictions of crystal stability. Nat. Comm..

[33] Blöchl PE (1994). Projector augmented-wave method. Phys. Rev. B.

[34] Kresse G, Joubert D (1999). From ultrasoft pseudopotentials to the projector augmented-wave method. Phys. Rev. B.

[35] Kresse G, Furthmüller J (1996). Efficiency of ab-initio total energy calculations for metals and semiconductors using a plane-wave basis set. Comput. Mater. Sci..

[36] Kresse G, Furthmüller J (1996). Efficient iterative schemes for *ab initio* total-energy calculations using a plane-wave basis set. Phys. Rev. B.

[37] Perdew JP, Burke K, Ernzerhof M (1996). Generalized gradient approximation made simple. Phys. Rev. Lett..

[38] Dudarev SL, Botton GA, Savrasov SY, Humphreys CJ, Sutton AP (1998). Electron-energy-loss spectra and the structural stability of nickel oxide: An LSDA + U study. Phys. Rev. B.

[39] Mosey NJ, Liao P, Carter EA (2008). Rotationally invariant *ab initio* evaluation of Coulomb and exchange parameters for DFT + U calculations. J. Chem. Phys..

[40] Singhal, S. C. & Kendall, K. High-temperature solid oxide fuel cells: fundamentals, design and applications (Elsevier, 2003).

[41] Lee J, Ohba N, Asahi R (2018). Discovery of zirconium dioxides for the design of better oxygen-ion conductors using efficient algorithms beyond data mining. RSC Adv..

[42] Lany S, Zunger A (2008). Assessment of correction methods for the band-gap problem and for finite-size effects in supercell defect calculations: Case studies for ZnO and GaAs. Phys. Rev. B.

[43] Lany S, Zunger A (2009). Accurate prediction of defect properties in density functional supercell calculations. Modell. Simul. Mater. Sci. Eng..

[44] Togo A, Tanaka I (2018). Spglib: a software library for crystal symmetry search. arXiv.

[45] Togo A, Oba F, Tanaka I (2008). First-principles calculations of the ferroelastic transition between rutile-type and CaCl_2_-type SiO_2_ at high pressures. Phys. Rev. B.

[46] Togo A, Chaput L, Tanaka I, Hug G (2010). First-principles phonon calculations of thermal expansion in Ti_3_SiC_2_, Ti_3_AlC_2_, and Ti_3_GeC_2_. Phys. Rev. B.

[47] Baroni S, Giannozzi P, Testa A (1987). Green’s-function approach to linear response in solids. Phys. Rev. Lett..

[48] Momma K, Izumi F (2011). VESTA 3 for three-dimensional visualization of crystal, volumetric and morphology data. J. Appl. Crystallogr..

