# Supplementary material for: Oxygen conduction mechanism in Ca3Fe2Ge3O12 garnet-type oxide
Source: Sci Rep. 2019 Feb 22;9:2593. doi: 10.1038/s41598-019-39288-x (PMC6384878; doi:10.1038/s41598-019-39288-x)
Supplement: Supplementary file 1 — Supplement [file 41598_2019_39288_MOESM1_ESM.pdf]

Supplementary Information

Oxygen conduction mechanism in $Ca_3Fe_2Ge_3O_{12}$ garnet-type oxide

Joohwi Lee[1], Nobuko Ohba[1], & Ryoji Asahi[1]

[1]Toyota Central R&D Laboratories, Inc., Nagakute, Aichi 480-1192, Japan

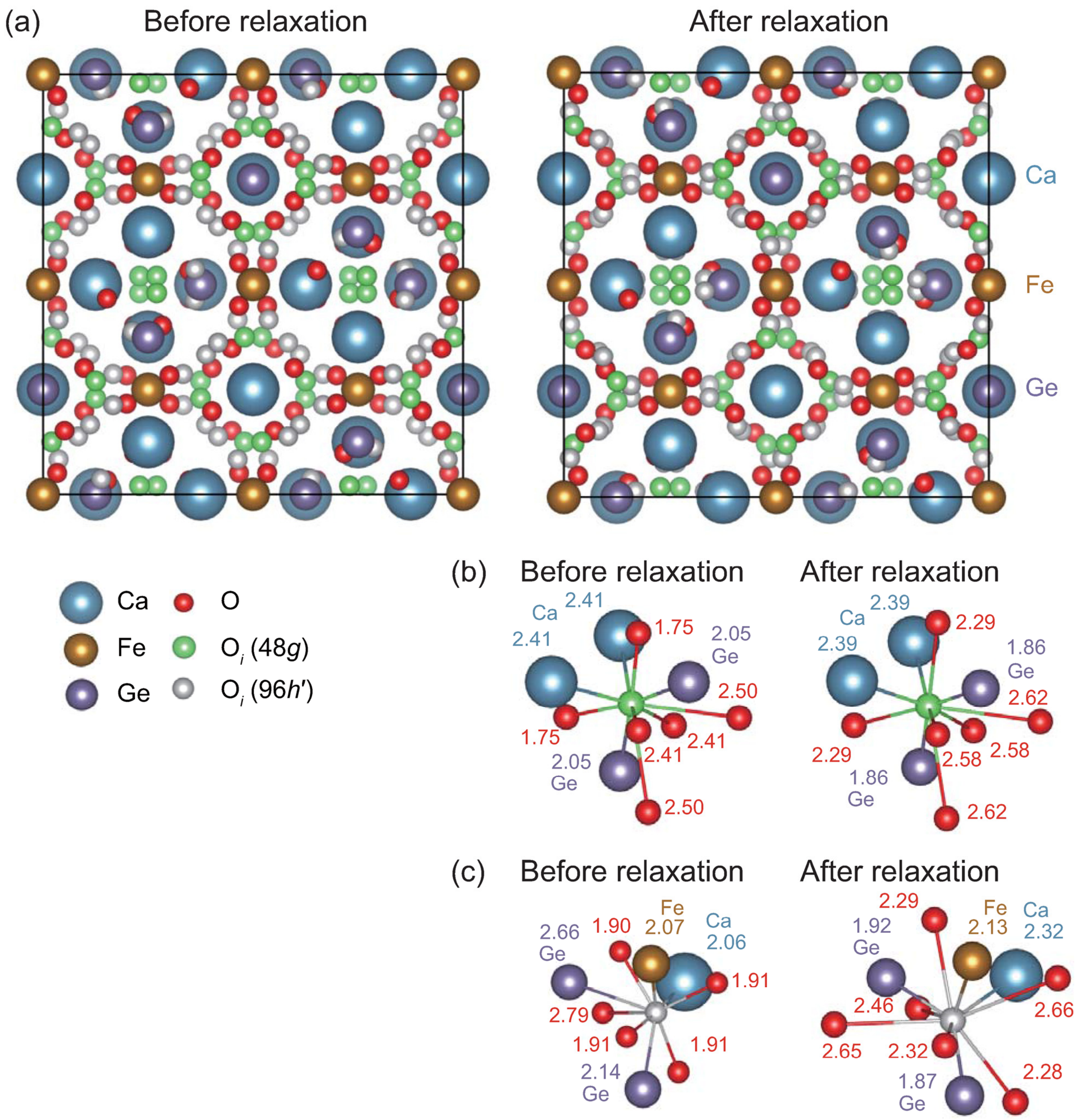

Figure S1. (**a**) Distribution of equivalent 48g and 96h' sites before and after the optimization of the internal coordinates with one doubly charged O_i. The internal coordinates of the cations and O are equal to those of the perfect crystal; only the changes in the internal coordinates of O_i are shown in the optimized cells. In the calculations, one O_i is incorporated in the computational cell ($Ca_{24}Fe_{16}Ge_{24}O_{96}$, 160 atoms). Nearest-neighboring atoms of (**b**) O_i in the 48g site and (**c**) O_i in the 96h' site before and after the optimization of the internal coordinates with one O_i. The numerical values are the distances (bond lengths), expressed in Å. The cutoff radius for the nearest-neighboring atoms in the figure is 2.8 Å, which is ~10% longer than the longest Ca–O bond.

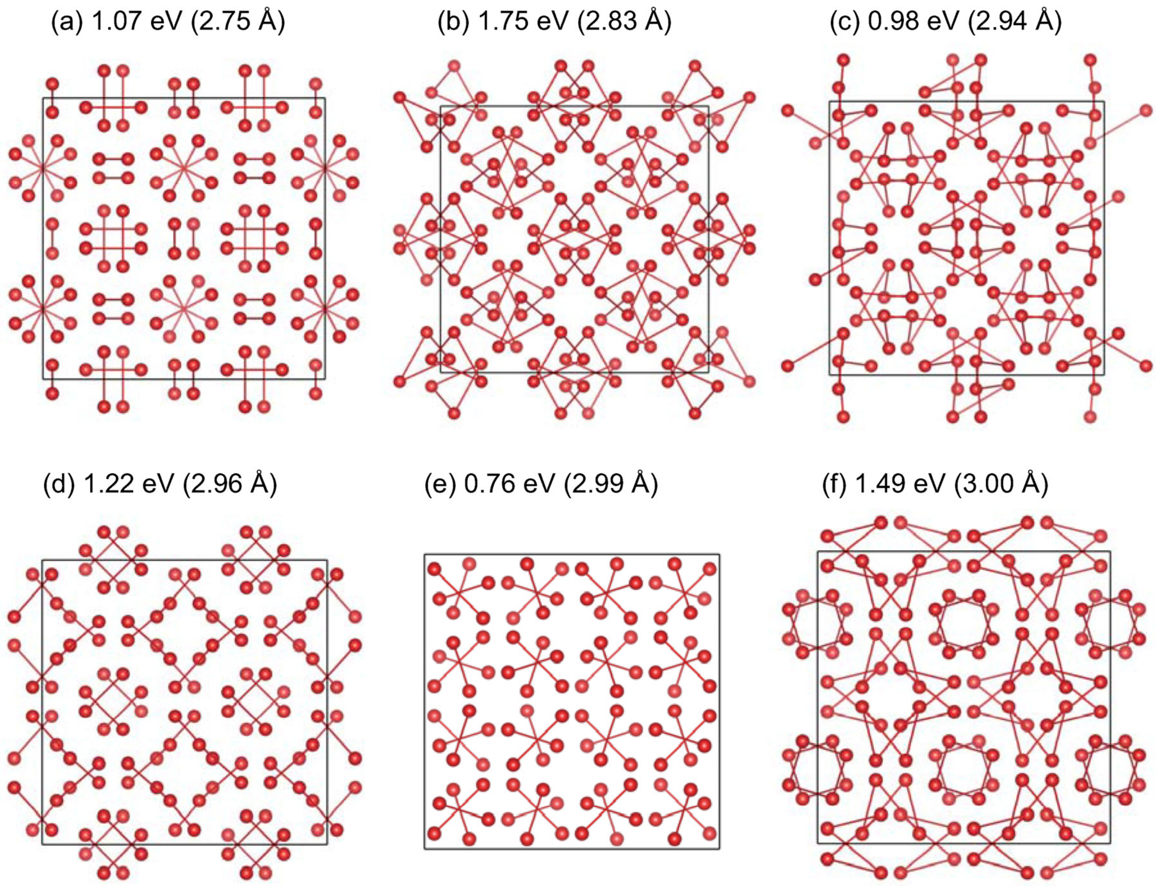

Figure S2. Initial setting for the calculations of migration paths considered as candidates for E_{mig} of V_O between V_O sites (= O sites, red spheres) in the garnet-type $Ca_3Fe_2Ge_3O_{12}$ viewed along the <100> direction. For an easier understanding of the migration paths, only V_O sites are shown. The numerical values are the distances between V_O sites in the unrelaxed cell and E_{mig} values. V_O is doubly charged. All of the migration paths are localized, so that several combinations of migration paths are required for V_O for the diffusion in the whole cell. The *a*, *b*, and *c* axes of the perfect crystal are equivalent to each other.

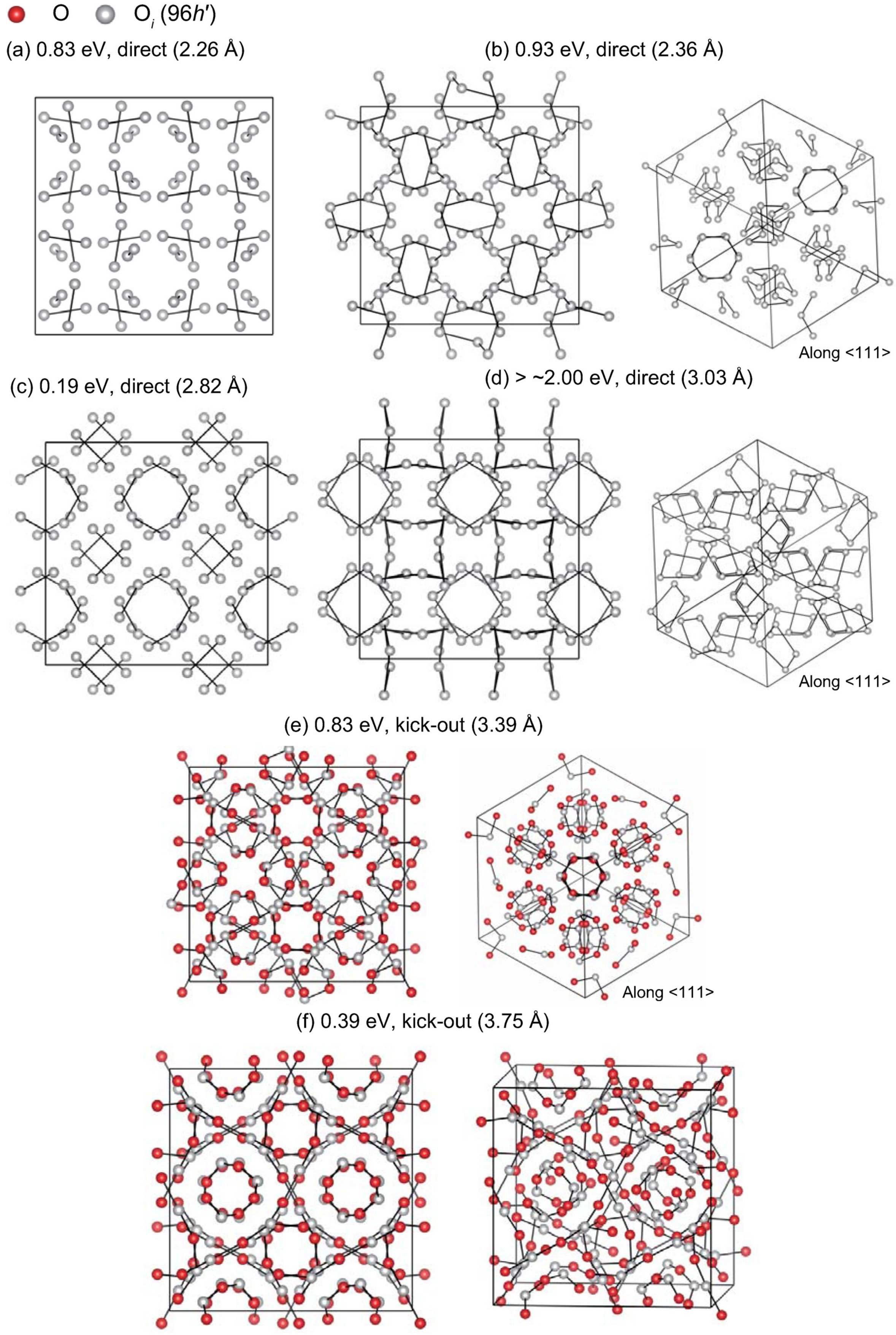

O
O_i (96h′)
(a) 0.83 eV, direct (2.26 Å)
(b) 0.93 eV, direct (2.36 Å)
Along <111>
(c) 0.19 eV, direct (2.82 Å)
(d) > ~2.00 eV, direct (3.03 Å)
Along <111>
(e) 0.83 eV, kick-out (3.39 Å)
Along <111>
(f) 0.39 eV, kick-out (3.75 Å)

Figure S3. Initial settings for the calculations of migration paths considered as candidates for E_{mig} of O_i between two 96*h*′ sites in the garnet-type $Ca_3Fe_2Ge_3O_{12}$ viewed along the <100> direction. Views along the <111> direction are also presented. For an easier understanding of the migration paths, only 96*h*′ for O_i or O sites (in the case where the kick-out mechanism is used) are shown. The numerical values are the distances between 96*h*′ sites in the unrelaxed cell and E_{mig} values. O_i is doubly charged. For (e) and (f), the CI-NEB calculations are performed using the migration path with the kick-out mechanism. The migration paths in (c) and (f) are referred to as 96*h*′–48*g*–96*h*′ and 96*h*′–O–96*h*′ migration paths in the main text, respectively. Only the migration path in (f) is delocalized for the whole cell, whereas the migration paths in (a)–(e) are localized.

(a)

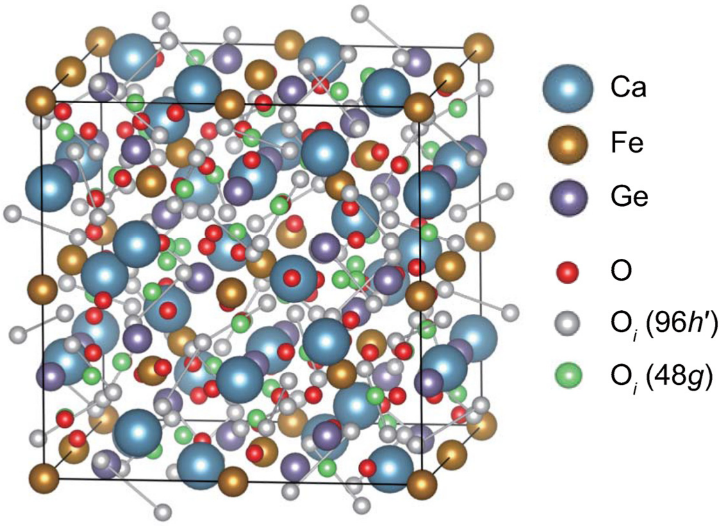

(b)

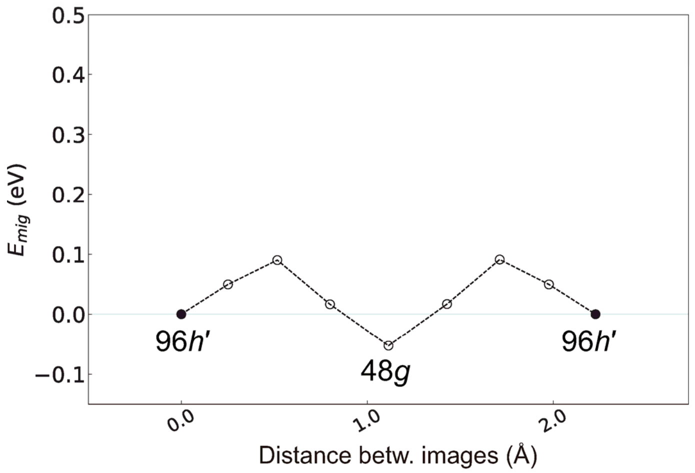

Figure S4. (**a**) Initial setting for the calculation of the 96h'–48g–96h' migration path [shown in Fig. S3(c)] with the 48g sites in the garnet-type $Ca_3Fe_2Ge_3O_{12}$. (**b**) CI-NEB profile of the O_i migration on this migration path obtained using the initial and final states with O_i in the 96h' sites. O_i is doubly charged. This migration path is almost identical to the localized 48g–96h' migration path. The closed circles denote the initial or final states fixed in the CI-NEB method.

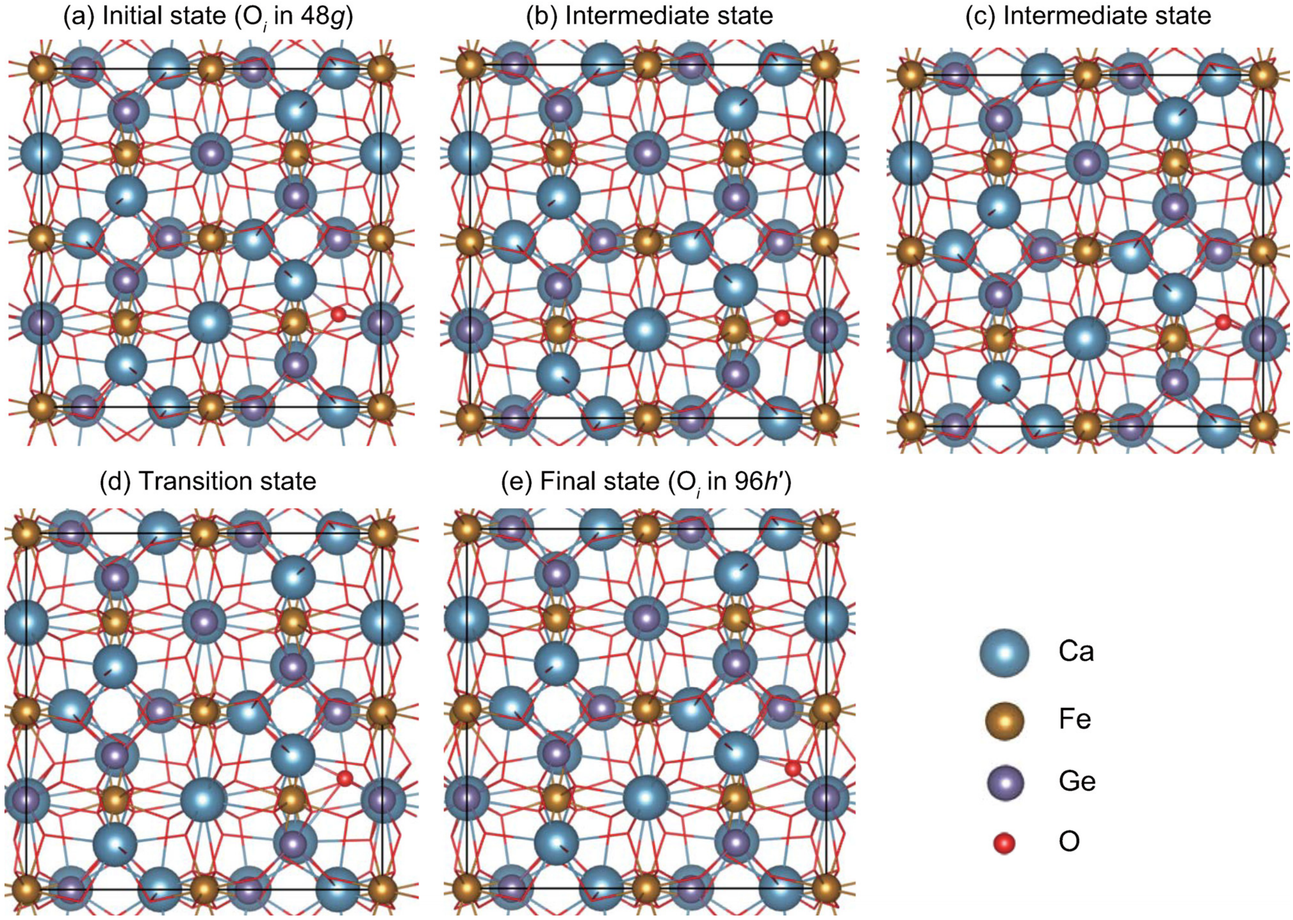

Figure S5. Snapshots of the 48*g*–96*h*′ migration path for O_i migration in the garnet-type $Ca_3Fe_2Ge_3O_{12}$ obtained by the CI-NEB method. Only the cations and moving O atoms are presented as spheres. O_i is doubly charged.

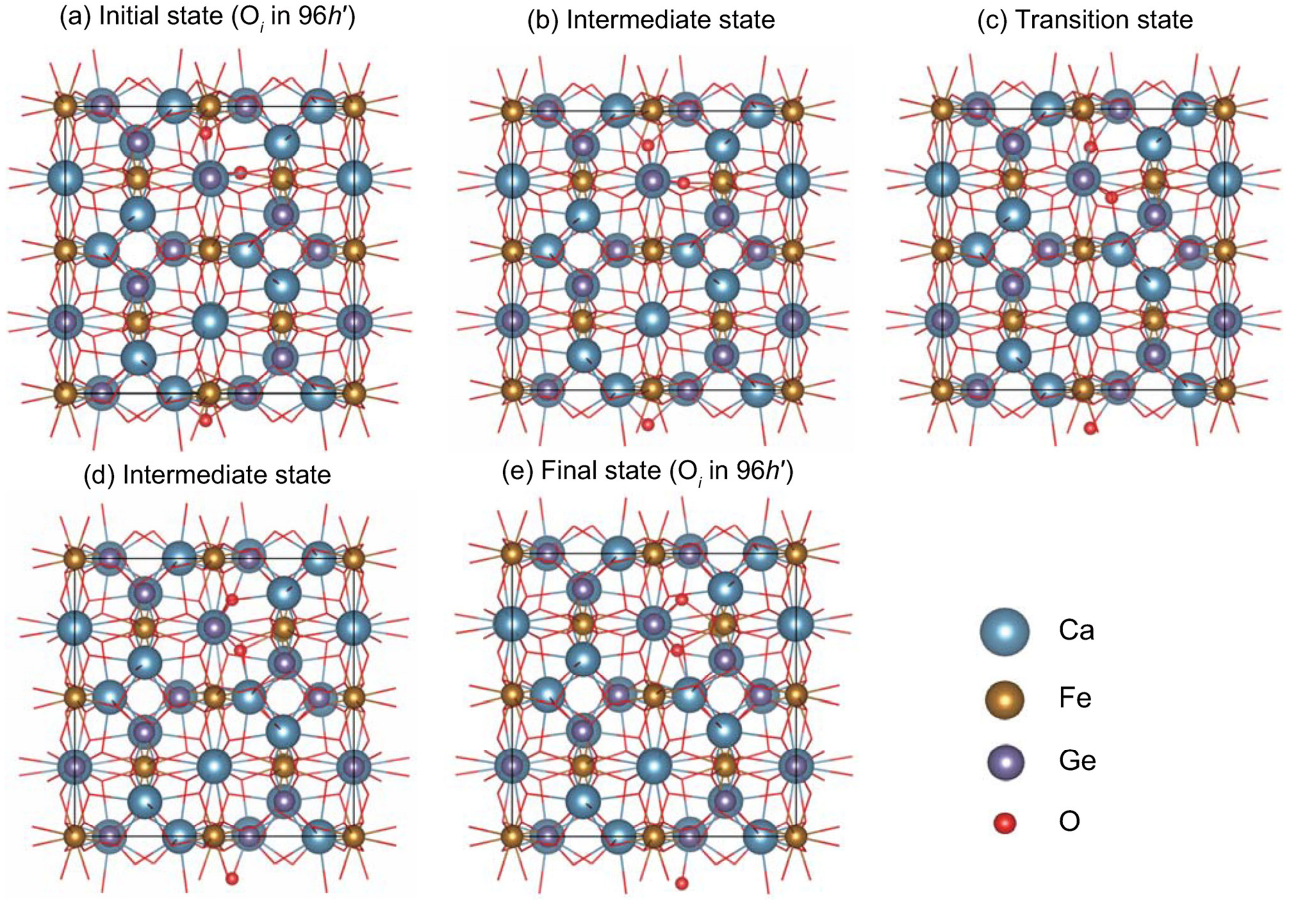

Figure S6. Snapshots of the 96h'–O–96h' migration path for O_i migration in the garnet-type $Ca_3Fe_2Ge_3O_{12}$ obtained by the CI-NEB method. Only the cations and moving O atoms are presented as spheres. O_i is doubly charged.

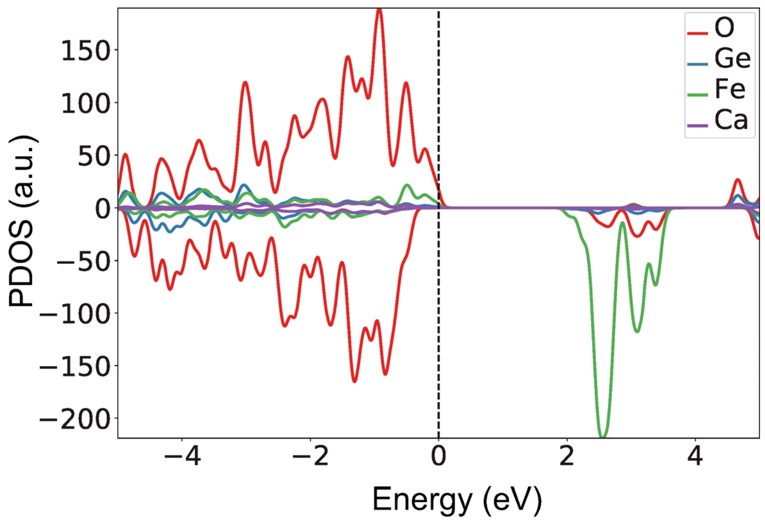

Figure S7. Projected electron density of states of $Ca_3Fe_2Ge_3O_{12}$ obtained using the GGA+U method. The zero energy corresponds to the Fermi level in the computation. The HOMO and LUMO are formed mainly by O 2*p* and Fe 3*d*, respectively.

Table S1. Formation energies of the antisites in $Ca_3Fe_2Ge_3O_{12}$ (160 atoms), calculated as differences in energies between the supercells with and without the antisites.

Antisite (exchanged ions)	Energy increase (eV)
Ca_{Fe} + Fe_{Ca}	2.26
Ca_{Ge} + Ge_{Ca}	4.29
Fe_{Ge} + Ge_{Fe}	1.09
Ca_O + O_{Ca}	6.56
Fe_O + O_{Fe}	Unstable [a]
Ge_O + O_{Ge}	5.50

[a] Fe and O atoms in O and Fe sites move to Fe and O sites to obtain the perfect crystal during the optimization of the internal coordinates.
